# Geospatial Heterogeneity of Hidradenitis Suppurativa Searches in the United States: Infodemiology Study of Google Search Data

**DOI:** 10.2196/34594

**Published:** 2022-06-09

**Authors:** Vishnutheertha Kulkarni, Ginette A Okoye, Luis A Garza, Shannon Wongvibulsin

**Affiliations:** 1 University of Queensland Medical School Brisbane Australia; 2 Department of Dermatology Howard University Hospital Washington DC, DC United States; 3 Department of Dermatology Johns Hopkins University School of Medicine Baltimore, MD United States; 4 Department of Biomedical Engineering Johns Hopkins University School of Medicine Baltimore, MD United States

**Keywords:** hidradenitis suppurativa, infodemiology, internet, digital dermatoepidemiology, epidemiology, big data, dermatology

Although hidradenitis suppurativa (HS) is a debilitating skin disease, clear epidemiology of HS is incomplete due to difficulties in data collection [1]. Infodemiology, the utilization of web-based data such as Google Trends for public health purposes, offers a potential solution [2]. With the use of online searches for health information, Google Trends offers a rich data source to address the challenges of population-level HS research [3]. Given that HS is a disorder of disparities [4], we hypothesize that there would be nonuniform HS search interest across the United States.

Relative search volume (RSV) data for Google searches using the keyword “hidradenitis suppurativa” were obtained with the following parameters: *United States*, *January 1, 2016 - December 31, 2021*, *all categories*, and *web search*. RSV data are scaled from 0 to 100, where 100 corresponds to the highest RSV. State-level RSV was normalized by search interest for “hidradenitis suppurativa” relative to all searches in that particular state during the time span of interest. The US geographic distribution of HS searches was visualized with a choropleth map. The heterogeneity of state-level RSV for “hidradenitis suppurativa” was compared with that of “skin” and “acne,” which are expected to have a more uniform distribution of searches. The Levene test was used to assess variance heterogeneity. R software (version 3.6.3; The R Foundation) was used for data analysis.

The heterogeneity of “hidradenitis suppurativa” searches is shown in Figure 1. The corresponding SD for the state-level “hidradenitis suppurativa” RSV was 13.8. In contrast, the SD for the state-level “skin” and “acne” RSV were 6.1 and 7.3, respectively. There was significant heterogeneity in the variance of “hidradenitis suppurativa” searches compared with that of both “skin” *(P*<.001) and “acne” (*P*<.001) searches.

We conclude that there are large geographic variations in HS searches that are not observed for skin or acne. Although a lack of population-level data on HS prevalence limits the ability to confirm whether “hidradenitis suppurativa” search heterogeneity is reflective of differences in the state-level prevalence of HS, prior work has demonstrated the correlation of Google search volume with cancer incidence [5]. Given the difficulty of data collection for population-level HS research, further exploring publicly available, real-time data from Google can offer a convenient way to examine the disparities associated with HS. A limitation is that different portions of the population utilize Google to varying extents and may not provide representative estimates for HS interest.

Our study presents insights into HS distribution and the potential for precision public health efforts to address areas with increased “hidradenitis suppurativa” searches that may be correlated with higher HS burden. This research provides the groundwork for using publicly available data as surveillance tools that can provide insights specific to populations of interest and offers a general methodological framework applicable to the investigation of dermatological diseases with challenging data collection. Overall, this big data digital dermatoepidemiological approach serves as an important foundation for further public health efforts and epidemiological studies on HS and health care disparities.

**Figure 1:**
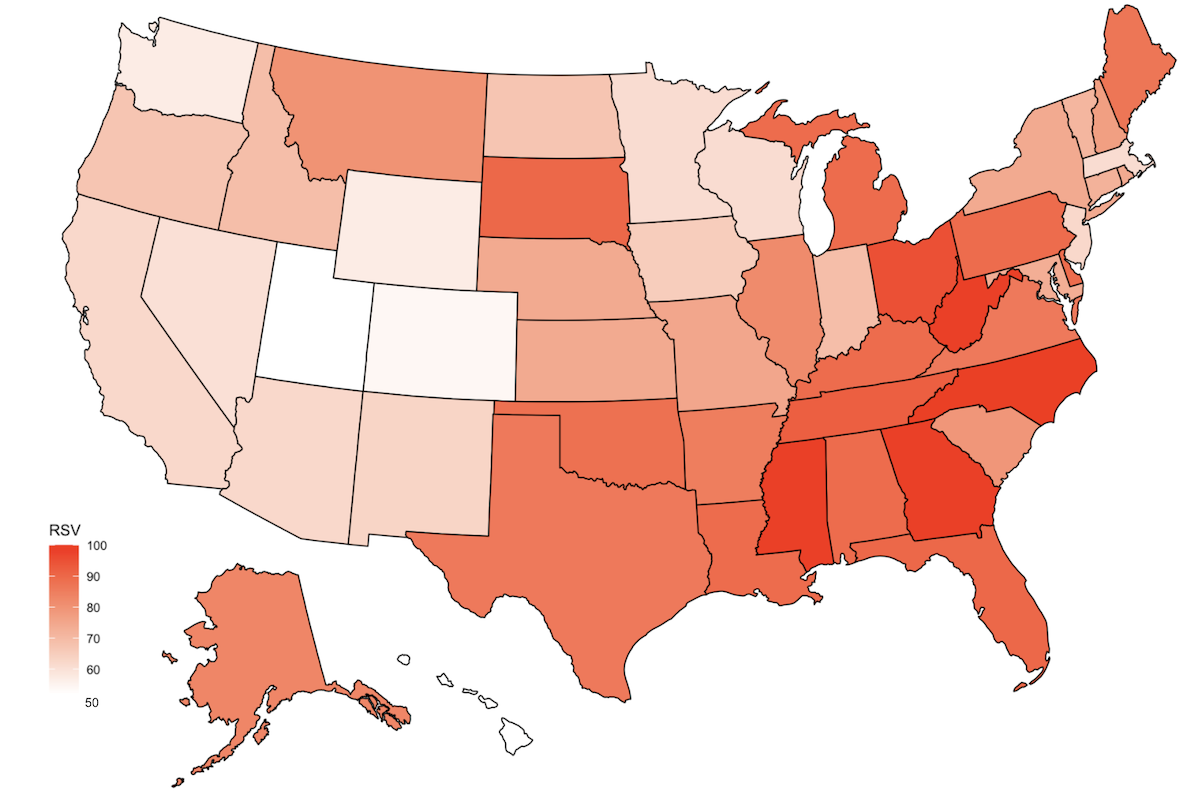
Choropleth map displaying the geographic distribution of search interest for the search term “hidradenitis suppurativa” through state-level relative search volume (RSV) data from January 1, 2016, to December 31, 2021, where dark red corresponds to the highest RSV and light red corresponds to the lowest RSV.

## Abbreviations

HS: hidradenitis suppurativa
NIH: National Institutes of Health
RSV: relative search volume
